# The First London *X*-Ray Convention

**Published:** 1909-04-10

**Authors:** 


### THE FIRST LONDON X-RAY CONVENTION.

A Convention to include all branches of medical electricity will be held for the first time in London from July 5 to 9, 1909, under the presidency of Dr. H. Lewis Jones, of St. Bartholomew's Hospital, and the vice-presidency of Mr. W. Deane Butcher. The inaugural meeting, the general meetings, the demonstrations, and the exhibition will be held at University College, Gower Street. The exhibition will include all classes of electrical and physical apparatus for medical treatment, and will be held simultaneously with the convention. Makers and inventors will have an opportunity of showing novelties in apparatus. Representatives of American and Continental Governments will be invited to take part in a discussion as to the best means of providing apparatus and training for the army and navy. Papers (limited to 15 minutes in duration of delivery) and debates will be in English, but papers in French and German will also be accepted provided *résumés* are sent in English. Various distinguished continental medical electricians, radiographers, and physicists have already promised to attend and read papers. The inclusive subscription for members of the convention will be one guinea, to be devoted to an entertainment fund. All medical practitioners are invited to attend the exhibition, the demonstrations, and the meetings, and to take part in the discussions, to which they will be admitted on presentation of their visiting cards. The executive committee consists of Dr. Lewis Jones, Mr. Dean Butcher, and Dr. E. Reginald Morton (honorary secretary and treasurer), and the organising secretary of the convention is Mr. Ernest Schofield, 11 Chandos Street, Cavendish Square, W., to whom all communications should be addressed.

